# *Momordica charantia*, a Nutraceutical Approach for Inflammatory Related Diseases

**DOI:** 10.3389/fphar.2019.00486

**Published:** 2019-05-08

**Authors:** Massimo Bortolotti, Daniele Mercatelli, Letizia Polito

**Affiliations:** ^1^Department of Experimental, Diagnostic and Specialty Medicine-DIMES, Alma Mater Studiorum, University of Bologna, Bologna, Italy; ^2^Department of Pharmacy and Biotechnology, Alma Mater Studiorum, University of Bologna, Bologna, Italy

**Keywords:** *Momordica charantia*, bitter melon, bitter gourd, natural products, nutraceuticals, anti-inflammatory agents, anti-cancer agents

## Abstract

*Momordica charantia*, commonly called bitter melon, is a plant belonging to Cucurbitaceae family known for centuries for its pharmacological activities, and nutritional properties. Due to the presence of many bioactive compounds, some of which possess potent biological actions, this plant is used in folk medicine all over the world for the treatment of different pathologies, mainly diabetes, but also cancer, and other inflammation-associated diseases. It is widely demonstrated that *M. charantia* extracts contribute in lowering glycaemia in patients affected by type 2 diabetes. However, the majority of existing studies on *M. charantia* bioactive compounds were performed only on cell lines and in animal models. Therefore, because the real impact of bitter melon on human health has not been thoroughly demonstrated, systematic clinical studies are needed to establish its efficacy and safety in patients. Besides, both *in vitro* and *in vivo* studies have demonstrated that bitter melon may also elicit toxic or adverse effects under different conditions. The aim of this review is to provide an overview of anti-inflammatory and anti-neoplastic properties of bitter melon, discussing its pharmacological activity as well as the potential adverse effects. Even if a lot of literature is available about bitter melon as antidiabetic drug, few papers discuss the anti-inflammatory and anti-cancer properties of this plant.

## Traditional Uses of *Momordica charantia*

*Momordica charantia* L. (MC), also known as bitter melon or bitter gourd, belongs to Cucurbitaceae family and grows in tropical and sub-tropical regions. The fruits and leaves of *Momordica* species are rich in phytochemicals and may have many health-promoting effects by offering nutritional and nutraceutical components. The plant has been known for ages and it has been used in many traditional and folk medicines (Polito et al., 2016a) for a wide range of medical applications, including the treatment of T2DM, hypertension, obesity, cancer, bacterial and viral infections, and even AIDS (Grover and Yadav, 2004). In Ayurveda medicine, bitter melon, known as karela, has been used for thousands of years. Its pharmacological properties are attributed to each part of the plant, i.e., seeds, roots, leaves, and particularly the unripe fruits (Scartezzini and Speroni, 2000). The juice found application for the treatment of many disorders: for example, it is used for joint pain relief and against chronic fever, in cases of jaundice and illnesses of the liver or the digestive system because of its diuretic, laxative and anti-helminthic actions. It is applied locally in case of chronic skin diseases and to treat burns, boils, and rashes. The use of the whole plant as food is recommended for the treatment of T2DM (Scartezzini and Speroni, 2000). In Turkish folk medicine the oil obtained from the ripe fruits of bitter melon, macerated in olive oil warmed by the sun, was combined with honey, and used for the prevention and healing of gastric ulcers (Gürdal and Kültür, 2013). In African folk medicine bitter melon is mainly used for worm infections, inflammation (fruits, seeds, and leaf juice), fever, menorrhea (leaves), syphilis, rheumatism, and skin diseases (roots). Leaf decoction is used in T2DM patients; fruits and leaves are used for the treatment of jaundice and other liver diseases and to cure ulcers and burns. Moreover, *Momordica* preparations are given for the treatment of gonorrhea, measles, chicken pox, scabies and malaria. In the Caribbean area, it is administered as a leaf decoction or fruit juice for the treatment of diabetes. The leaf decoction is also used for the treatment of high blood pressure, womb infections, malaria, dysentery, and worm infections. Leaf baths are used for rheumatism therapy (Polito et al., 2016a).

## Chemical Constituents and Nutritional Value of *Momordica charantia*

The major chemical constituents of MC are classified as: (i) heteropolysaccharides, mainly composed of galactose, glucose, arabinose, rhamnose, and mannose; (ii) proteins and peptides, such as momordins, momorcharins, MAP30 and MC lectin, belonging to the ribosome-inactivating proteins family (RIPs) (Schrot et al., 2015); (iii) terpenoids and saponins, such as cucurbitanes and cucurbitacines; (iv) flavonoids and phenolic compounds; (v) other compounds such as essential oils, fatty acids, amino acids, and sterols (Dandawate et al., 2016). Chemical structures of the main bioactive MC constituents are reported in Supplementary Figure S1.

Nutritional analysis demonstrated that this plant possesses the highest nutritive value among cucurbits, being a good source of carbohydrates, proteins, fibers, vitamins, and minerals. Fruits are composed by 93.2% of water, while protein and lipids account for 18.02 and 0.76% of its dried weight, respectively (Saad et al., 2017). Green fruits contain vitamin C, A and P, thiamine, riboflavin, niacin, and minerals (Gupta et al., 2011). In addition, MC seeds can represent a good source of lipids, such as polyunsaturated fatty acids (nearly 45% of the weight) and they are among the few foods containing conjugated linolenic acid, being 63–68% as eleostearic acid (Yoshime et al., 2016). The essential oil, obtained from drought seeds, contains sesquiterpenes, phenylpropanoids and monoterpenes. Other bioactive compounds, such as tocopherols and polyphenols have been reported in MC seed oil (Nyam et al., 2013). The pericarp, the aril, the stem and the leaves of the plant are also a good source of phenolic compounds, which can be useful to protect from oxidative damage by acting directly on reactive oxygen species and to induce endogenous defense systems (Yoshime et al., 2016).

Several glycosides isolated from MC fruit and stem have been grouped as cucurbitane-type triterpenoids, being cucurbitacins the main ones. They exhibit a broad range of biological activities, mainly anti-inflammatory and anti-diabetic (Rios et al., 2005).

## Anti-Inflammatory and Anti-Oxidant Activity of *Momordica charantia*

Lifestyle and dietary habits contribute to a chronic state of low-grade inflammation, which can alter immune status and gut microbiota. Various dietary components have the potential to modulate predisposition to chronic inflammatory conditions and can be helpful in their therapy. Nevertheless, the relationship among most of these dietary components and their anti-inflammatory mechanisms is unclear (Minihane et al., 2015).

*Momordica charantia* dietary supplementation has been widely studied to treat several diseases, like T2DM, dyslipidemia, obesity and cancer, thus showing that MC extracts possess hypoglycemic and lipid-lowering properties, even if clinical trials conducted so far gave inconclusive results (Alam et al., 2015). In diabetic patients, the chronic systemic inflammation contributes to increase blood glucose concentration and represents a risk factor in developing cardiovascular diseases and obesity. Chronic inflammation is involved in the pathogenesis of different diseases: a clear association has been established for neurodegenerative diseases, obesity, metabolic syndrome, cardiovascular disease, T2DM, and cancer (Minihane et al., 2015). Several evidences indicate that oxidative stress plays a role in chronic inflammatory diseases. Thus, oxidative stress and inflammation are closely related pathophysiological processes that can activate each other (Biswas, 2016). MC beneficial properties seem dependent on its anti-inflammatory and anti-oxidant activities (Chao et al., 2014; Dandawate et al., 2016). Various MC extracts were found to regulate inflammation mainly through NF-κB signaling pathway inhibition: in RAW 264.7 cells, bitter melon reduced TNF-α production, induced by LPS, decreasing the expression of LPS-induced inflammatory genes, including those for IL-1α, IL-1β, and TNF-α. The MC extracts also reduced NF-κB DNA binding activity and phosphorylation of p38, JNKs, ERKs as well as MAPKs (Kobori et al., 2008a). Moreover, MC showed reduction of LPS-induced NO and prostaglandin E2 production together with a reduction of inducible NO synthase and IL-1β expression (Lii et al., 2009). In the same cell model, a dose-dependent inhibition of NO production for MC extract was demonstrated (Svobodova et al., 2017); but it was also reported that MC extracts reduced expression levels of inducible NO synthase and cyclooxygenase-2, suppressing NF-κB, and activator protein-1 (AP-1) activity via downregulation of ERKs and Akt (Hsu et al., 2013; Yang et al., 2018). The effects of a triterpene purified from bitter melon was investigated against TNF-α-induced inflammation via AMP-activated protein kinase in FL83B cells. This compound suppressed the TNF-α-induced expression of inflammatory markers, including inducible NO synthase, p65 subunit of NF-κB, TNF-α, and IL-1β (Cheng et al., 2012). In C57BL/6 mice fed with high-fat diet supplemented with MC, a decrease in serum C reactive protein and IL-6 concentrations together with a loss of hyperglycemia and hyperlipidemia was reported (Xu et al., 2014). The MC-containing diet also normalized serum levels of the cytokines suggesting its role in reducing inflammation, obesity and insulin resistance in obese mice (Bao et al., 2013). Dietary supplementation with MC powder in high-fat diet obese mice was showed to lower systemic inflammation by reducing TNF-α and IL-6 serum levels and to remodel key functions of colon by altering transcriptomic profile and affecting the expression of genes involved in the regulation of inflammation (Bai et al., 2016, 2018). Recently, it was reported that MC extracts reduced intercellular adhesion molecule-1 expression and upregulated mir-221/-222 in TNF-α treated lung tissues in mice, also decreasing PI3K/Akt/NF-κB/IκB. MC extracts were given before TNF-α, suggesting that bitter melon supplementation may be useful as a chemo-preventive agent in individuals at risk for inflammatory-related diseases (Sung et al., 2018). Therefore, MC exerts its anti-inflammatory effects by acting on several important signaling pathways involved in inflammation.

The potential anti-oxidant activity of MC extracts has been evaluated in several *in vitro* studies. Pretreatment of neuroblastoma cells with MC extracts was found to attenuate cytotoxic oxidative stress induced by H_2_O_2_ by increasing intracellular scavenger activity and reducing H_2_O_2_-induced activation of the JNKs, p38, and ERK1/2 MAPK signaling pathways (Kim et al., 2018). MC fruit extract significantly reduced neuro-inflammation, ameliorating the consequent neurodegenerative diseases (Nerurkar et al., 2011). Xanthine oxidase is a key enzyme for the induction of hyperuricemia and gout and it is involved in many inflammation related diseases, such as metabolic syndrome, and in augmented cancer risk (Battelli et al., 2016, 2018). Cucurbitane-type triterpene glycosides isolated from MC stems and fruits significantly inhibit xanthine oxidase activity (Lin et al., 2012). Triterpenoids isolated from MC stems have also shown scavenging activities and inhibitory effect on xanthine oxidase activity (Liu et al., 2010). Anti-oxidant compounds in bitter gourd pulp and seed powders showed potential natural anti-oxidant activity to inhibit the lipid peroxidation (Padmashree et al., 2010). Moreover, MC was evaluated for its anti-oxidant activity *in vitro* showing an amelioration of oxidative damage induced by peroxynitrite (Kim et al., 2013). It has been demonstrated that, after blanching, bitter gourd considerably decreases its phenolic content and anti-oxidant activity (Myojin et al., 2008). Therefore, the MC anti-oxidant activity seems dependent both from direct radical scavenge of MC components and from their effect on oxidant enzymes.

Bitter melon extracts were also found to alleviate bacterial-induced inflammation. For example, MC extracts reduced *Propionibacterium acnes*-induced skin inflammation in mice and suppressed the cytokine and matrix metalloproteinase-9 levels in *Propionibacterium acnes*-induced inflammation of THP-1 cells. This activity was attributed to the anti-inflammatory effects of phenolic compounds present in the extract (Huang et al., 2015). Cucurbitane triterpenods isolated from MC leaves strongly suppressed *Porphyromonas gingivalis*-induced IL-8, IL-6, and IL-1β levels (Tsai et al., 2016).

The anti-inflammatory activity of MC supplementation has been also demonstrated in patients with primary knee osteoarthritis, in a single-blinded, randomized trial. Thirty-eight patients were daily treated for 3 months with commercially available MC supplementation. After 3 months, there were significant improvements in knee osteoarthritis and reduction in analgesic score; also, body weight, body mass index, and fasting blood glucose were significantly reduced. In this study, it was demonstrated that MC can represent an alternative to reducing pain and improving symptoms among patients while reducing the need for analgesic drug consumption (Soo May et al., 2018).

**FIGURE 1:**
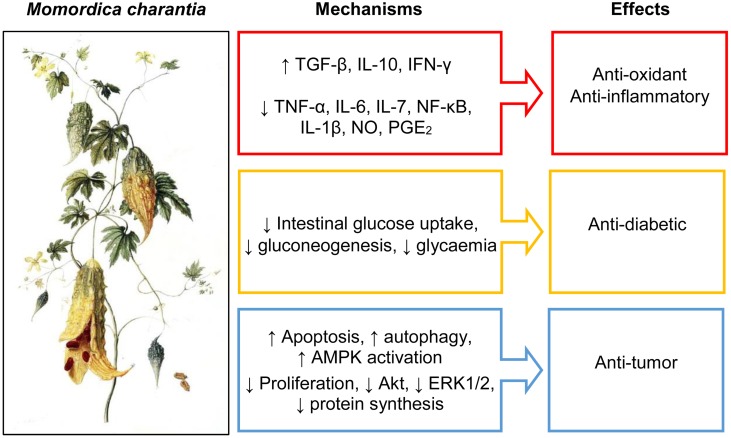
Main mechanisms of *Momordica charantia* pharmacological effects.

The main mechanisms of MC anti-inflammation and anti-oxidant actions are summarized in Figure 1. In Table 1 we summarized some of the main *in vitro* and *in vivo* studies conducted so far to investigate MC anti-inflammation and anti-oxidant actions.

**Table 1:** Main anti-oxidant and anti-inflammatory studies carried out *in vitro* and *in vivo* with MC components.

Pathological process	Compound(s)	*In vitro*	Animals	References
Inflammation	MC extract		Mice	Deng et al., 2019
Oxidative stress	MC ethanol extract	SK-N-MC cells		Kim et al., 2018
Oxidative stress	MC leaf ethanol extract		Obese mice	He et al., 2018
Inflammation	MC extracts		ApoE KO C57BL/6 mice	Zeng et al., 2018
Inflammation	MC powder		Obese Sprague-Dawley rats	Bai et al., 2016, 2018
Inflammation	MC fruit extracts	A549 cells	C57BL/6 mice	Sung et al., 2018
Oxidative stress/inflammation	Bioactive peptide BG-4	THP-1 cells		Jones et al., 2018
Oxidative stress/inflammation	MC methanol extract	RAW264.7 cells		Lii et al., 2009; Yang et al., 2018
Oxidative stress/inflammation	MC polysaccharides	Oxidative stress cell free assays Primary neuronal cells	Gastritis/myocardial infarction/ischemia-reperfusion/rat models	Gong et al., 2015; Tan and Gan, 2016; Raish, 2017; Raish et al., 2018
Inflammation	MC fruit juice	T-cells	Diabetic Wistar rats	Fachinan et al., 2017a,b
Oxidative stress/inflammation	MC extract		Holstein-Friesian cows	Emre et al., 2017
Oxidative stress	MC anthocyanins	Oxidative stress cell free assays		Güdr, 2016
Oxidative stress	MC extract	Oxidative stress cell free assays		Padmashree et al., 2010; Ghous et al., 2015; Aljohi et al., 2016
Oxidative stress	MC metanolic, ethanolic and butanolic extracts	Oxidative stress cell free assays		Yadav et al., 2016
Inflammation	MC leaf extract	Porphyromonas gingivalis-induced THP-1 stimulation	Mice skin inflammation model	Huang et al., 2015; Tsai et al., 2016
Inflammation	MC fruit and seeds extract		BALB/c mice with sepsis	Chao et al., 2014; Ciou et al., 2014
Inflammation	MC fruit ethyl acetate extract	RAW 264.7 cells		Hsu et al., 2013
Inflammation	MC fruits		Obese C57BL/6 mice	Bao et al., 2013
Inflammation	Cucurbitane-type triterpene	RAW 264.7 cells; FL83B cells		Cheng et al., 2012; Liaw et al., 2015
Oxidative stress/inflammation	MC aqueous extracts		Obese mice; Diabetic rats	Tripathi and Chandra, 2010; Xu et al., 2014
Inflammation	Butanol extract	RAW 264.7 cells		Kobori et al., 2008a


## Anti-Cancer Activity of *Momordica charantia*

Accumulating evidence shows that chronic inflammation can promote tumor initiation and malignant progression of many cancers. Two pathways linking inflammation and cancer are generally identified: (i) tumor-extrinsic inflammation, which is caused by many factors, including bacterial and viral infections, lifestyle and exposure to environmental pollutant, and it is mediated by innate immunity cells; (ii) tumor-intrinsic inflammation, which is due to neoplastic mutations leading to the production of inflammatory mediators and the recruitment of immunity cells in the tumor microenvironment, contributing in an inflammatory milieu promoting several steps of cancer progression. These two pathways share common features, such as the production of primary inflammatory cytokines like IL-1, IL-6, and TNF-α and the activation of known transcription factors involved in the regulation of inflammatory response, such as NF-κB and STAT3 (Marelli et al., 2017). Considering the importance of inflammatory changes in different cancer types, preventing or reversing inflammation has become an important approach to control neoplasia progression.

In Table 2 we summarized some of the main *in vitro* and *in vivo* studies conducted so far to investigate MC anti-cancer activity.

**Table 2:** Main anti-tumoral studies carried out *in vitro* and *in vivo* with MC components.

Tumor	Compound(s)	Cells	Animals	References
Breast	Cucurbitane-type triterpene	MCF-7, MDA-MB-231		Weng et al., 2013
	RNase MC2	MCF-7		Fang et al., 2012a
	MC extract	MCF-7, MDA-MB-231		Ray et al., 2010
	Eleostearic acid	MDA-ERα7		Grossmann et al., 2009
	Water MC extract		SHN mice	Nagasawa et al., 2002
	MAP30	MDA-MB-231	SCID mice	Lee-Huang et al., 2000
	α-momorcharin	MCF-7, MDA-MB-231, MDAMB-453	Balb/C mice	Cao et al., 2015
Colon	Methanol MC extract	HT-29, SW480, HFF		Kwatra et al., 2013
	Methanol MC extract	Hone-1, AGS, HCT-116, CL1-0		Li et al., 2012a
	Acid and alkali MC extracts	SGC-7901		Li et al., 2012b
	MAP30	LoVo		Fan et al., 2008
	MC fatty acids	Caco-2		Yasui et al., 2005
	MC extract		Swiss mice	Deep et al., 2004
	MC extract		F344 rats	Kohno et al., 2004
Liver	MAP30	Hep G2	Balb/C nude mice	Fang et al., 2012b
	RNase MC2	Hep G2	Balb/C nude mice	Fang et al., 2012c
	MC lectin	Hep G2, PLC/PRF/5	Nude mice	Zhang et al., 2015
	Cucurbitane-type triterpene glycosides	Hep G2, Hep 3B		Yue et al., 2019
Prostate	MC extract	PC3, LNCaP	TRAMP mice	Ru et al., 2011
	MC leaf extract, Kuguacin J	LNCaP, PNT1A		Pitchakarn et al., 2011
	MC leaf extract	PLS10	Nude mice	Pitchakarn et al., 2010
	MCP30	LNCaP, PC-3, RWPE-1, PIN	Nude mice	Xiong et al., 2009
Bladder	48–127/momordin IT	T24		Battelli et al., 1996
Glioma	Transferrin/momordin IT	HS683, U251		Gosselaar et al., 2002
Lymphoma	Ber-H2/momordin IT	ALCL	SCID mice	Terenzi et al., 1996
	OM124/momordin IT	Daudi, EHM, BJAB, Raji, BM21	SCID mice	Bolognesi et al., 1998
Leukemia	Ethanol MC extracts	ED, Su9T01, S1T, HUT-102, MT-2, Jurkat, MOLT-4		Kai et al., 2011
	Ethanol MC extracts	HL60	Balb/cAnNCrj-nu/nu mice	Kobori et al., 2008b
	Anti-CD5/momordin IT	Peripheral blood mononuclear cells, Jurkat	nu/nu mice	Porro et al., 1993


Several phytochemicals, including MC extracts, are described to possess promising potentials as adjuvants in conventional anticancer therapies, due to their ability to prevent cancer progression (Salehi et al., 2018). MC extracts have been investigated mainly for their potential use as chemo-preventive agents; many studies have evaluated the efficacy of MC extracts or purified components against different tumor derived cells, suggesting that dietary consumption of MC could help to lower risk of several cancers. Anti-proliferative and immunomodulatory effects were reported in the majority of studies (Nerurkar and Ray, 2010). It is thought that MC extracts’ anti-cancer properties could rely on the ability to modulate several de-regulated signaling pathways in different type of cancer, like MAPK pathway, Akt/mTOR/p70S6K pathway through activation of AMPK, Wnt/β-catenin signaling pathway and through the modulation of cell cycle proteins, thereby inducing cell cycle arrest or inducing apoptosis or other cell death pathways. Three recently identified MC cucurbitane-type triterpene glycosides showed significant anti-tumor activity in hepatic carcinoma derived cell lines (Yue et al., 2019). Given the influence of MC extracts on several inflammatory-related signaling pathways, it is possible that MC anti-inflammatory properties may play a major role in its efficacy as tumor-preventive agent (Farooqi et al., 2018). To date, anti-cancer activity has been only observed in cancer cell lines and xenografted mice, and there is a need of further studies to elucidate the possible use of MC extracts as nutraceuticals in the treatment of cancer. A significant anti-tumor activity was reported for some MC proteins belonging to RIP family (Bolognesi et al., 2016). These proteins are potent inhibitors of cell translation and they have been extensively used for the production of anti-cancer drugs, particularly in the form of immunoconjugates or ITs, to obtain selective toxic protein delivery to target malignant cells (Polito et al., 2016b).

Although the lack of clinical data demonstrating the anti-tumor effect of MC components on humans, the whole results available in the literature make highly plausible a protective effect of MC both in the initiation of the tumor cell and during tumor progression. It is well known that the initial neoplastic transformation can be favored by oxidative stress that could be prevented by MC components. Tumor progression toward malignity is strongly related to chronic inflammation that is responsible for tumor invasion of surrounding normal tissues and angiogenesis. Again, the MC components could exert their anti-tumor effects by modulation of the inflammation status. The main mechanisms of MC anti-tumor action are summarized in Figure 1.

## Safety of *Momordica charantia*

Despite the wide MC usage in several traditional medicine, mainly for T2DM, there are quite scarce data from clinical trials and the few published studies enrolled a limited number of patients, for these reasons, safety data are more often derived from animal models. This lack of standardization in clinical studies still represents a limitation in the recognition of the therapeutic value of MC by a part of the scientific community. MC efficacy and safety have been comprehensively described by Basch et al. (2003). A recent meta-analysis highlighted the scarcity of data from clinical trials and the need for more structured and well-conducted studies (Peter et al., 2019).

*Momordica charantia* drugs should be always avoided by subjects that reported allergy to other plants from Cucurbitaceae. Individuals with glucose-6-phosphate dehydrogenase deficiency can develop favism after MC consumption (Raman and Lau, 1996). People wishing to procreate should consider with caution the daily use of MC, as it strongly reduced fertility in animal models (Stepka et al., 1974). As well as great caution should be used in the consumption of MC during pregnancy, since proteins contained in MC extracts showed abortive properties in animals (Basch et al., 2003). Caution is required also in patients with liver disease, because transaminase augment, although without histopathological alterations, was reported in animals. The most serious adverse effects in humans were reported in two small children that experienced a strong reduction of glycaemia after drinking MC based beverage on an empty stomach: between 1 and 2 h after ingestion, the children had convulsions followed by hypoglycemic coma (Raman and Lau, 1996). Other data on pediatric dosages are lacking.

A case of acute interstitial nephritis was reported in a 60-years male with T2DM and hypertension that used hyponidd, an ayurvedic drug containing *M. charantia*. The patient took one hyponidd tablet daily for 1 week before the onset of his symptoms: edema and a decrease in urine, which progressed to complete anuria in 2–3 days (Beniwal et al., 2017). The toxicity for kidney was already reported in mice treated with MC 4 g/kg for more than a week (Mardani et al., 2014).

## Conclusion

About two third of world population continue to prefer local folk medicine to industrial drugs, mainly for economic reasons but in many cases also because of the fidelity to a traditional life style. The interest in plant derived medicinal products is increasing also in the occidental countries because these kinds of drugs are considered more “natural” and thus less toxic (often erroneously). Bitter melon is commonly used as a natural drug, mainly for the treatment of T2DM, but also as anti-inflammatory and anti-oxidant. It is well known the MC contribute in lowering glycaemia in T2DM patients. Moreover, the above reported researches attesting the MC anti-inflammatory effects seem to indicate the possible contribute of MC preparations also to reduce analgesic drug consumption in inflammatory related diseases.

However, it should be considered that the variability of preparation and the differences in cultivar and plant variety, the stage of harvest, the part of plant used and other factors can contribute to the discordant findings across the literature and often make difficult to fix the optimal dosage in terms of both efficacy and safety.

In addition, some considerations should be taken into account about MC side effects and interaction with conventional drugs: (i) for patients assuming MC at high doses and for prolonged periods clinical signs attributable to renal distress should not be underestimated; (ii) caution should be used when MC is taken together with other blood glucose-reducing agents because of the possible additive effects.

In conclusion, more trials are necessary to establish MC efficacy and safety with the aim to better utilize this precious natural resource.

## Author Contributions

All authors collected the literature, wrote, and revised the manuscript.

## Conflict of Interest Statement

The authors declare that the research was conducted in the absence of any commercial or financial relationships that could be construed as a potential conflict of interest.

## Supplementary Material

Figure S1 Chemical structures of the main bioactive *Momordica charantia* constituents.

## Abbreviations

ERK: extracellular signal-regulated kinases
IL: interleukin
IT: immunotoxin
JNK: c-Jun N-terminal kinases
LPS: lipopolysaccharide
MAP30: *Momordica* anti-viral protein of 30 kDa
MAPKs: mitogen-activated protein kinases
MC: *Momordica charantia*
NF-κB: nuclear factor kappa-light-chain-enhancer of activated B cells
NO: nitric oxide
RIP: ribosome-inactivating-protein
T2DM: type 2 diabetes mellitus
TNF-α: tumor necrosis factor-alpha
