# Supplementary figures and images for: Momordica charantia, a Nutraceutical Approach for Inflammatory Related Diseases
Source: Front Pharmacol. 2019 May 8;10:486. doi: 10.3389/fphar.2019.00486 (PMC6517695; doi:10.3389/fphar.2019.00486)

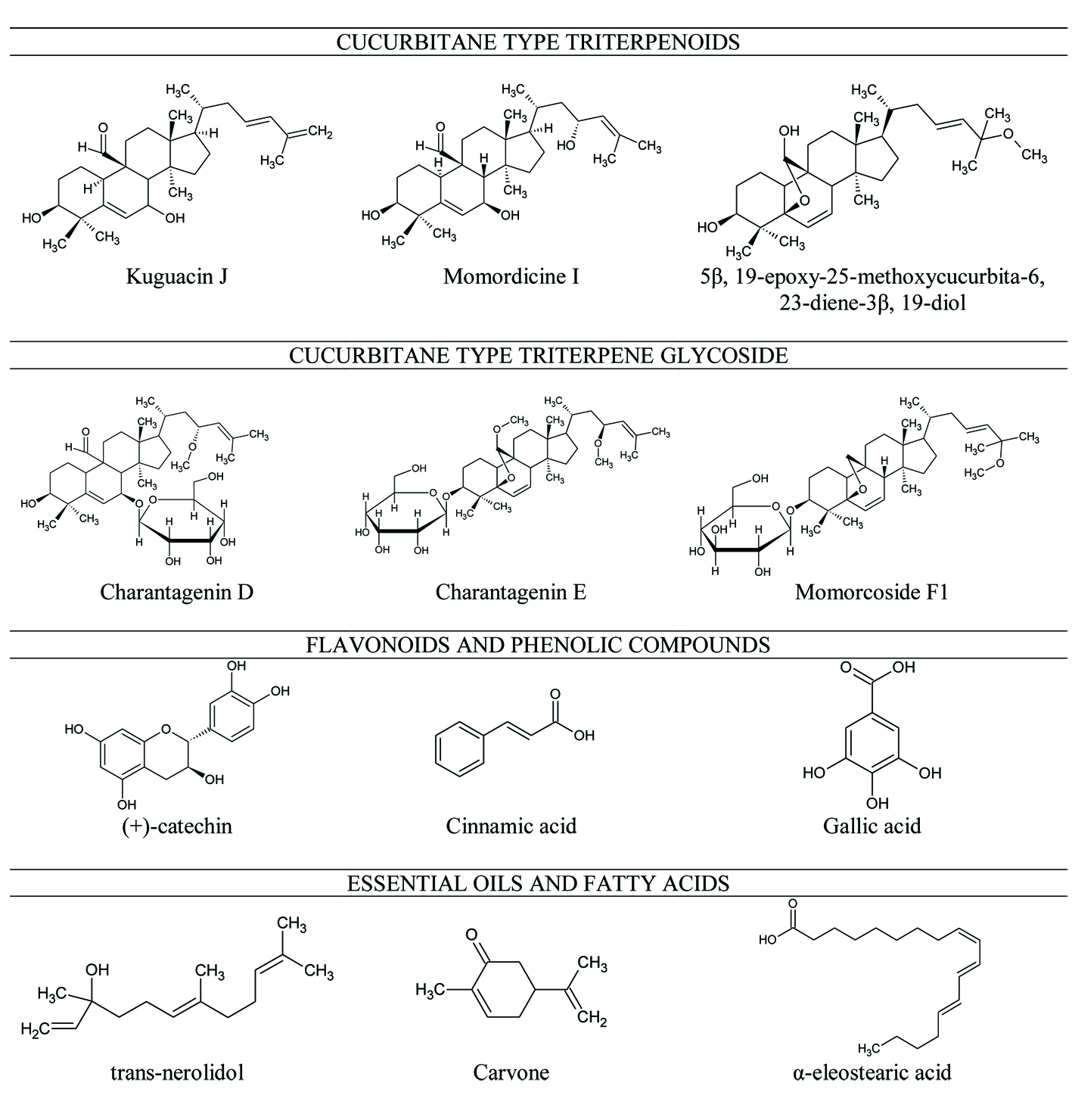

Supplement: Figure S1 — Chemical structures of the main bioactive Momordica charantia constituents. [file Image_1.JPEG]
